# Integrating Neurology and Psychiatry throughout Educational Curricula for Healthcare Professionals

**DOI:** 10.33696/neurol.1.026

**Published:** 2020

**Authors:** Brinda Desai Bradaric, Alana E Kirby, T. Celeste Napier

**Affiliations:** 1 Department of Health Sciences, Rush University Medical Center, Chicago, IL 60612, USA; 2 Department of Neurological Sciences, Rush University Medical Center, Chicago, IL 60612, USA; 3 Department of Psychiatry and Behavioral Sciences, Rush University Medical Center, Chicago, IL 60612, USA; 4 Center for Compulsive Behavior and Addiction, Rush University Medical Center, Chicago, IL 60612, USA

**Keywords:** Impulse control disorder, Neurology education, Psychiatry education, Interdisciplinary communication, Medical curriculum

We recently reviewed the scientific literature linking dopamine agonist pharmacotherapy for neurological disorders to the development of impulsive and compulsive spectrum disorders (ICSDs) [1]. This link was not clinically recognized until thousands of treated patients suffered from devastating emotional, financial and social difficulties associated with the co-occurring addictions. Here, we expand on this scientific overview to comment on how the clinical scenario emerged, and educational solutions to avoid similar consequences in the future. In brief, we hold that bridging the brain-centric disciplines (e.g., neurology and psychiatry) within medical education curricula and training is key. Teaching of these disciplines to future health professionals needs to emphasize integrated learning and practice to improve patient care.

Medical education and training on diagnosis of brain dysfunction and pharmacological interventions tends to be separated by field of study. For example, the pathophysiology, symptomology and treatment of Parkinson’s disease (PD) are taught in neuroscience courses. Such diseases are diagnosed by neurologists using motor symptoms. Psychosocial behavioral disorders and treatment are taught in psychology and psychiatry courses and are characterized by descriptions of behavior and mental status. This separation reflects the historical tendency for neurologists and psychiatrists to view brain dysfunction differently [2,3]. PD is a brain disease associated with pathology or anatomical changes in neurotransmitter substrates of particular brain regions (e.g., lesions in the dopaminergic neurons of the basal ganglia). By contrast, psychiatrists refer to mental health disorders as functional dysregulation of diffuse brain systems. Neurologists and psychiatrists have differing clinical philosophies to treating diseases, and the medical language used to describe brain dysfunction also differs in the two fields. For example, neurologists describe dysfunction by emphasizing the specific substrate damage or pathology (e.g., dopamine dysregulation syndrome), whereas psychiatrists emphasize mental function within the overall brain (e.g., substance use disorder). Though it may seem practical to separate diseases that afflict the brain and mind, these traditions and philosophies do not emphasize the substantial overlap between traditional neurologic and psychiatric manifestations. The separation also has implications for treating clinicians, as comorbid disorders are often mismanaged, under recognized and under treated [4–6]. Symptoms and treatment side effects that lie at the intersection of neurology and psychiatry may be missed or neglected by either discipline individually [2].

Development of ICSDs in PD patients following dopamine agonist therapy is an excellent example of a condition that exists at the intersection of neurology and psychiatry. The basic science and clinical evidence that link ICSDs in PD is detailed in Napier et al., 2020 [1] and includes: (i) onset of ICSDs is related to initiation of, or the increase in dose for, dopamine agonist therapy, and (ii) reducing the dose of the dopaminergic agonist or terminating treatment reduces or terminates ICSDs. In 2010, a large cross-sectional study of 3,090 idiopathic parkinsonian patients in the USA and Canada reported a high prevalence of ICSD during dopamine agonist therapy [7]. This landmark study was a collaboration among psychiatrics and neurologists, and its publication is highly recognized as a pivot point in raising clinical awareness of the ICSD risks of dopamine agonist therapy [8–15]. We consider that clinical awareness should have long-preceded 2010, as the function of dopamine agonists and their cognate receptors have been known for decades. To make this point, a brief overview of dopamine pharmacology is provided.

Dopamine agonists have high affinity for, and are efficacious at, dopamine receptors. Apomorphine was first recognized as a dopamine agonist in the 1960s. As PD was known to reflect deficits in brain dopamine transmission in brain regions that govern motor function, apomorphine was clinically explored as a PD therapeutic. However, clinical use was limited by side effects and the utility of the dopamine precursor, levodopa (L-DOPA) in conjunction with peripherally acting carboxylase inhibitor (e.g. carbidopa) became appreciated. L-DOPA/carbidopa provides excellent relief of motor symptoms of PD, but the side effect profile includes problematic fluctuations in motor benefits, and with chronic use, many patients develop troublesome dyskinesias. Ergot-alkaloid dopamine agonists, such as bromocriptine, pergolide and cabergoline, were developed to combat the side effects and complications associated with chronic levodopa therapy [16,17]. The use of these drugs was expanded to early PD, driven in part by the desire to spare patients the adverse effects of L-DOPA. However, the ergot-derived agonists have a high risk of cardiac valvular, pulmonary and peritoneal fibrosis [9]. During the 1990’s non-ergot alkaloids, e.g., pramipexole and ropinirole, were developed to selectively target particular subtypes of dopamine receptors and avoid the side effect profiles seen with receptor non-selective ergot-alkaloid dopamine agonists. In 1997, pramipexole and ropinirole were approved for clinical use in the United States and there was a rapid increase in their clinical use.

Dopamine receptors are classified based on genetic and protein homology, and effects on signaling cascades. There are two major classes (or families), D1 and D2. The D1 family includes subtypes, referred to as D1 and D5. The D2 family includes D2, D3, and D4 subtypes. Pramipexole and ropinirole have particularly high affinity for the D2 and D3 subtypes. By contrast, the older agonists are less selective, activating receptors in both D1 and D2 families. The D2/D3 receptor preference affords a clinical profile wherein motor benefits are obtained, while avoiding the side effects often seen with dopamine agonists that activate both receptor families. However, D2/D3 receptors are not only localized to brain regions that regulate motor function, e.g., the basal ganglia, but are highly expressed through out limbic brain regions. It has long been known that limbic regions govern emotion-laden, reward-motivated behaviors. These regions remain largely intact in PD, and thus, D2/D3 receptor-preferring agonists ‘over activate’ the limbic system which can lead to ICSDs (overviewed in Napier et al., 2020) [1].

ICSDs that occur during dopamine agonist therapy can cause significant harm to patients. Behaviors such as gambling, drug abuse, and sexual promiscuity have destroyed the finances and relationships of patients on dopamine agonists [18,19]. Healthcare outcomes often are significantly worsened, including patient experience, effectiveness of care, increased time of care and management, and increased number of readmissions or office visits. The psychosocial behavioral side effects occur more frequently with dopamine agonists compared to other available treatments for PD [7]. Thus, earlier recognition of ICSDs during agonist therapy could have changed clinical practice and prevented patient harm.

To ensure that brain diseases and disorders that intersect neurology and psychiatry are recognized, managed and treated, students need to be trained to think of brain pathology and dysfunction in an integrated fashion. Our current educational system offers limited interaction between neurology and psychiatry. Currently, undergraduate education and training produce expert clinicians in their chosen discipline, but this model contributes to the persistence of specialty silos and limited crosstalk between fields. Identifying and addressing the link between dopamine agonists and ICSDs, for example, requires clinicians to connect neurobiological substrates, pharmacological effects, and psychiatric symptoms. Recognizing biologically plausible neuropsychiatric side effects requires integrating knowledge from the separate contexts of neurology and psychiatry. Ideally, these gaps in knowledge could be addressed with changes to the curriculum allowing topics to be introduced and taught in combination. Such training would enhance teamwork among clinicians, which is particularly important in complex neurological and psychiatric conditions, and patients would receive holistic treatment, which can improve health care outcomes [4–6,20–25].

There are many opportunities for interdisciplinary education on the brain in health profession curricula. For integration to be most impactful, it should be introduced during bachelor’s education, reinforced during medical education, and mastered during residency training. Bachelor’s degree course work lays the groundwork for the understanding of the human nervous system and behavior. However, many students who aspire to advanced healthcare practice degrees earn a bachelor’s degree without exposure to psychology, neuroscience or either field. According to the American Association of Medical Colleges, of the 21,869 students that matriculated into medical school in 2019, 57% majored in biological sciences and 1% in social sciences [26]. Students in these majors may be exposed to psychology and neuroscience as part of their curriculum. However, this is not a guarantee as biology majors at a 4-year university are not generally required to take psychology as a part of the major core requirements. Furthermore, the 42% of matriculants with alternate majors [26] likely have no requirement to take classes either in psychology or the neurosciences. The lack of basic background in neuroscience and psychology in the current educational system is the first stumbling block in training clinicians who are able to recognize the neurobiological substrates involved in disorders that lie at the intersection of neurology and psychiatry, such as ICSDs during dopamine agonist therapy for PD.

The four years of bachelor’s study can be leveraged with courses that allow students to make connections between the fields of neurology and psychiatry. Bachelor level education can include neuropsychology courses as an elective for future health care professionals. This requirement could be independent of the student’s major to increase awareness and learning of foundational concepts of complex brain disorders. An introductory course can provide the foundations of normal brain function and anatomy and its relation to behavior. Advanced courses in neuropsychology can focus on changes of structure and function in relation to changes to behavior, with theoretical and practical applications. Well-established neuroscience and psychology courses could incorporate lectures, case studies, or workshops that showcase the relationship between brain systems and behavior. By intentionally teaching the two subjects together, students can start to make the connections between brain function and behavior. These foundational courses can teach the student how to learn and integrate fundamental concepts in neurology and psychiatry.

To reinforce the fundamental concepts learned during bachelor’s education, medical education in professional schools must continue integrating neurology and psychiatry within the curriculum. Currently, in the United States, physicians complete four years of undergraduate medical education. During this education, medical students are largely exposed to psychological concepts separately from neurological concepts, and treatment of the mind separately from treatment of the body. This leaves newly minted clinicians ill prepared to appreciate connections between these two fields. To facilitate bridging concepts of neurology and psychiatry, the coursework in the first two years of medical undergraduate education can integrate normal and abnormal function of neurobiological substrates and pathways when discussing disease, mental function and clinical behavior. The relationship between neurology and psychiatry can be further developed as students learn about medications used to treat neurological and psychiatric diseases. This allows students to ascertain the similarities and differences between treatments and conceptualize potential neurological and psychiatric side effects that can be associated with therapy. Further, case studies that are used to provide a practical clinical application of the learning materials can include examples of disease and/or side effects from medications that intersect neurology and psychiatry. Integration of neurology and psychiatry allows for common concepts to be combined, which offsets the apparent increase in coursework by reducing repetition and facilitating learning of advanced and complex concepts. These changes to curriculum during the first years of medical school can help students master the connections between brain function and behavior. The last years of medical school can reinforce interdisciplinary learning by allowing those students who have chosen neurology or psychiatry as their track, to have rotations in child neurology and psychiatry, as well as electives in subspecialties such as neuroradiology, neuropathology, and geriatrics [27]. These rotations can foster interaction between neurology and psychiatry, reinforce the connection between brain function and behavior, and strengthen the concept of interdisciplinary collaboration for students.

For clinicians to master neurological and psychiatric concepts and apply them to their practice, exposure to both fields must continue during residency training and fellowship. Currently, an overwhelming majority of physicians in the United States complete one residency track, such as internal medicine, psychiatry or neurology. Out of 4 years of training, residents in psychiatry are required to spend up to 2 months on a clinical neurology rotation, and neurology residents spend up to 1 month on a psychiatry service. Though several medical institutions across the United States have combined neurology-psychiatry residency programs, the length of these programs can be unattractive. To address this need, medical centers can create dual residency programs that are limited to 4–5 years or limit rotations in one specific area. Having residents and fellows rotate through clinics, such as memory disorder clinics, epilepsy centers and geriatric clinics that may have patients that exhibit both neurological and psychiatric symptoms, are great experiences that allow trainees to work closely with members of both specialties. It can also allow a reduction in the number of rotations within either discipline. These rotations would provide a holistic experience with intentional cross-disciplinary training.

Integration of neurology and psychiatry that occurs throughout education (bachelor’s, medical) and training will lead to clinicians developing an expertise in brain diseases and behavior, as well as improved awareness and management. Repetition is the key to learning and improved practice. Further, repetition of integrated learning and team-based practice can take multiple forms, such as case studies, presentations/seminars, and grand rounds. The more often students are taught and trained in an integrated learning environment where they are forced to make connections and apply information to different fields, the more likely they will be to recognize diseases that intersect neurology and psychiatry. The more often students are placed in an environment that allows for collaboration, the more likely they will be to see the value of teamwork.

Though challenging, integrating neurology and psychiatry would be beneficial. Both neurology and psychiatry have robust and rigorous curricula, and any integration of the two fields will require careful consideration to maintain equity between them. Potential problems including duration of training and curricular scope will need to be taken into careful consideration. These challenges can be overcome and the outcome rewarding. An integrated education and training can produce clinicians with a better understanding of brain diseases/disorders and treatment, so they are better prepared to diagnose difficult and complex diseases that have clinical features of both neurology and psychiatry. Moreover, clinicians will be aware of side effects associated with pharmacological intervention and be able to critically evaluate diseases and medications that straddle neurology and psychiatry, such as the risk of developing ICSDs with dopamine agonists that we have highlighted in this commentary. Clinicians will be able to better counsel patients and caregivers about potential side effects associated with medications and clinical symptoms that may emerge due to diseases and/or disease progression. The complementary nature of neurology and psychiatry can help advance our knowledge of brain disease by provide biological basis for psychiatric disorders and more nuanced behavioral analysis for neurological disorders. Finally, integration within the educational and training system may potentially reduce the stigma associated with psychiatric illness. Ultimately, we believe that implementing these changes will improve individualized patient care and healthcare outcomes.
